# Plasma proteomic profiles correlate with organ dysfunction in COVID‐19 ARDS

**DOI:** 10.14814/phy2.70300

**Published:** 2025-04-02

**Authors:** Moemen Eltobgy, Brett Klamer, Daniela Farkas, James D. Londino, Joshua A. Englert, Jeffrey C. Horowitz, Rama K. Mallampalli, Guy Brock, Joseph S. Bednash

**Affiliations:** ^1^ Department of Internal Medicine, Division of Pulmonary, Critical Care, and Sleep Medicine The Ohio State University Columbus Ohio USA; ^2^ Dorothy M. Davis Heart and Lung Research Institute (DHLRI), College of Medicine, The Ohio State University Columbus Ohio USA; ^3^ Department of Biomedical Informatics The Ohio State University Columbus Ohio USA; ^4^ The Center for RNA Biology College of Medicine, the Ohio State University Columbus Ohio USA

**Keywords:** acute respiratory distress syndrome, COVID‐19, proteomic analysis, SARS‐CoV‐2

## Abstract

Severe COVID‐19 is often complicated by hypoxemic respiratory failure and acute respiratory distress syndrome (ARDS). Mechanisms governing lung injury and repair in ARDS remain poorly understood. We hypothesized that plasma proteomics may uncover protein biomarkers correlated with COVID‐19 ARDS severity. We analyzed the plasma proteome from 32 patients with ARDS and COVID‐19 using an aptamer‐based platform of 7289 proteins, and correlated protein measurements with sequential organ failure assessment (SOFA) scores at days 1 and 7 of ICU admission. We identified 184 differentially abundant proteins correlated with SOFA at day 1 and 46 proteins at day 7. In a longitudinal analysis, we correlated dynamic changes in protein abundance and SOFA between days 1 and 7 and identified 40 significant proteins. Pathway analysis of significant proteins identified increased ephrin signaling and acute phase response signaling correlated with increased SOFA scores between days 1 and 7, while pathways related to pulmonary fibrosis signaling and wound healing had a negative correlation. These findings suggest that persistent inflammation may drive disease severity, while repair processes correlate with improvements in organ dysfunction. This approach is generalizable to future ARDS cohorts for identification of biomarkers and disease mechanisms as we strive towards targeted therapies in ARDS.

## 1 INTRODUCTION

Severe COVID‐19 (coronavirus disease 2019) results in respiratory failure and the acute respiratory distress syndrome (ARDS), a form of hypoxemic respiratory failure characterized by diffuse lung inflammation and bilateral alveolar fluid accumulation that manifests radiographically as opacities on chest imaging (Matthay et al., 2024). Patients with ARDS account for 10% of ICU admissions with mortality rates ranging from 30% to 50% (Bellani et al., 2016). Most decedents from COVID‐19 show evidence of ARDS at autopsy (Elsoukkary et al., 2021; Schaller et al., 2020), and ARDS mortality dramatically increased during the COVID‐19 pandemic (Oud & Garza, 2023). ARDS may occur following a variety of insults and is known to be heterogeneous, with varying clinical courses, outcomes, and mechanistic drivers (Calfee et al., 2014, 2015; Reilly et al., 2014). There are no specific pharmacologic therapies for ARDS. Many research groups have hypothesized that ARDS encompasses many subgroups or subtypes with distinct pathobiological mechanisms. There is an ongoing need to better understand heterogeneity in ARDS to inform the development of biomarker‐directed, targeted therapeutics and individualized management of patients with ARDS.

Proteomics has the potential to advance our understanding of biological mechanisms and pathobiology in many diseases, including ARDS (Hanash, 2003; Jimenez & Verheul, 2014). Mass spectrometry approaches to proteomic analysis are costly, time‐consuming, and pose challenges for data analysis. In biological specimens, abundant proteins, such as albumin in blood samples, hinder resolution of less abundant proteins that may have important functions during disease (Ignjatovic et al., 2019). Transcriptomic studies are limited to a data layer that is one step removed from biologic function and may not accurately reflect levels of functional protein in different biologic cells, tissues, and fluids. This is highlighted by evidence suggesting that significant differences between the transcriptome and proteome arise (Dick et al., 2023; Gunawardana & Niranjan, 2013; Juschke et al., 2013; Takemon et al., 2021) due to extensive post‐transcriptional and post‐translational regulation in health and disease. The development of high‐throughput proteomics arrays that do not rely on mass spectrometry has increased speed, decreased cost, and allowed for reproducible detection of important protein biomarkers, rendering these approaches a desirable tool for drug discovery (Iwamoto & Shimada, 2018), disease prediction, and biomarker detection (Birhanu, 2023; Clarke et al., 2012; Srinivas et al., 2002). While previous studies have reported proteomic profiling for COVID‐19, few studies specifically addressed severe COVID‐19 and ARDS (Batra et al., 2022; Filbin et al., 2021; Patel et al., 2021).

In this study, we investigate the circulating proteome from human plasma collected from 32 patients with COVID‐19 ARDS at two different time points using banked samples from critically ill patients with ARDS on day 1 and day 7 of ICU admission. Using an aptamer‐based array (SomaScan® platform by SomaLogic, Inc.), we analyzed the abundance of 7289 proteins and correlated differential protein abundance with patient Sequential Organ Failure Assessment (SOFA) scores. SOFA scores are a validated scoring system in critically ill patients, used to assess and monitor organ dysfunction (Moreno et al., 1999; Vincent et al., 1996, 1998). The study aims to characterize the temporal changes in the circulating proteome and explore how plasma protein abundance correlates with SOFA score in COVID‐19 ARDS for insights into underlying mechanisms of the disease in COVID‐19 ARDS.

## 2 METHODS

### 2.1 Study design and sample collection

**FIGURE 1:**
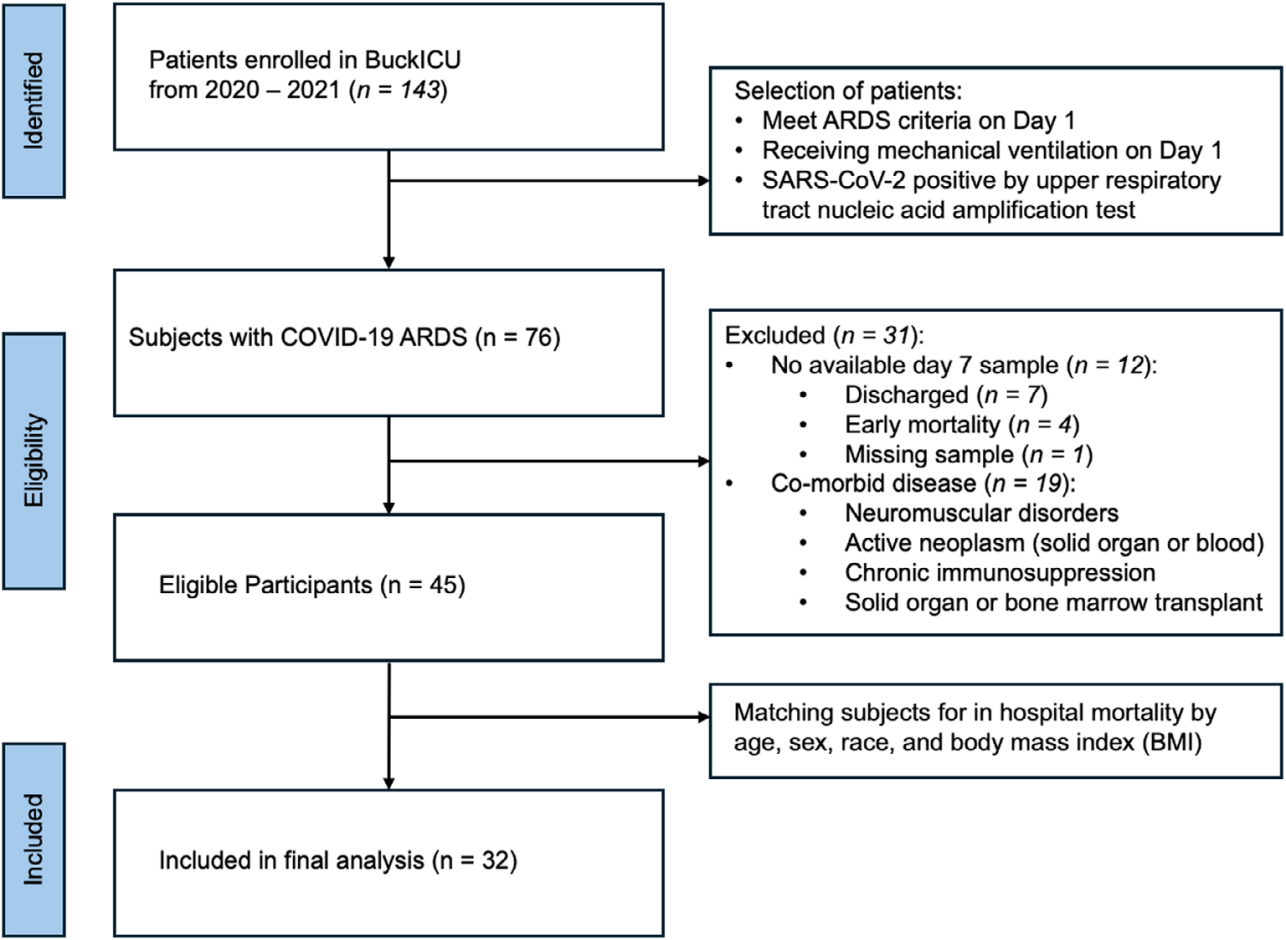
Schematic diagram of the cohort design. Patients with ARDS and COVID‐19 were identified from the BuckICU biorepository. Patients without available biologic samples and those with certain co‐morbid illnesses were excluded. The primary analysis was designed to compare differentially abundant proteins between survivors and non‐survivors, and 32 patients were included in the final analysis. Secondary analyses included all 32 patients and correlated differential protein abundance to SOFA scores.

Patients enrolled between May 2020 and June 2021 were identified from the Ohio State University Intensive Care Unit Registry (BuckICU), an IRB‐approved (IRB #2020H0175) biorepository that enrolls patients within 48 hours of admission to the intensive care units at the Ohio State University Wexner Medical Center and the Arthur G. James Cancer Hospital and Richard J. Solove Research Institute with acute respiratory failure and/or suspected sepsis. For inclusion in BuckICU, acute respiratory failure is defined by an increase in supplemental oxygen requirement to maintain oxygen saturation (SpO2) greater than 92% or the need for adjunctive respiratory support. Suspicion of sepsis is defined by meeting SIRS criteria (Levy et al., 2003) and clinical suspicion of infection (collection of any clinical culture specimen OR initiation of antibiotics). Following completion of the study protocol, diagnoses are adjudicated by two pulmonary and critical care physicians. While SIRS criteria were used for screening purposes, Sepsis‐3 guidelines were used to define sepsis during case adjudication. Sepsis‐3 defines sepsis as organ dysfunction caused by dysregulated host response to infection represented by an increase in Sequential Organ Failure Assessment (SOFA) score of at least 2 points due to infection (Guarino et al., 2023; Singer et al., 2016). At the time of sample submission, 143 patients were enrolled in BuckICU. For this study, we first identified 76 patients with ARDS as defined by the Berlin definition (Force et al., 2012) at Day 1, receiving mechanical ventilation, with a positive SARS‐CoV‐2 upper respiratory tract nucleic acid amplification test. We excluded 12 patients without available Day 1 and Day 7 blood samples and 19 patients with co‐morbid disease that may impact respiratory physiology or immune responses, specifically neuromuscular disease, active neoplasm, chronic immunosuppression, solid organ transplant, and bone marrow transplant. Our primary analysis was designed to identify differences in the plasma proteome between survivors and non‐survivors of COVID‐19 ARDS. These groups were matched by age, sex, race, and body mass index (BMI). A total of 32 patients were included in the analyses, as shown in Figure 1. Per BuckICU protocols, blood is collected in citrate vacutainer tubes, followed by immediate centrifugation to separate blood components. Plasma is stored as 500 microliter aliquots at −80 degrees C.

### 2.2 SomaScan protein quantification

To quantify protein abundance in human plasma, we used the SomaScan® 7 K assay v4.1 (SomaLogic, Inc), a DNA‐aptamer‐based array that measures over 7000 unique proteins. DNA aptamers, or SOMAmer® reagents, are chemically modified short sequences of DNA, designed for protein affinity, large dynamic range, and slow off‐rate. SomaScan 7 k is a validated tool used to characterize protein abundance in biologic samples in a variety of diseases, including blood‐based proteomic studies of pulmonary disease (Suryadevara et al., 2024) and cancer (Gupta et al., 2023). We provided one plasma aliquot without prior freeze/thaw for each patient and time point for analysis. Prior to analysis, raw data undergoes several normalization and calibration steps, including (1) hybridization normalization where each microarray is scaled according to the signal of 12 control aptamers to account for technical variability in individual microarrays, (2) intraplate median signal normalization, (3) plate scaling, (4) calibration of each aptamer to the median value observed in Calibrator Control samples, and (5) adaptive normalization to a reference using Adaptive Normalization by Maximum Likelihood (ANML) to adjust for inter‐sample variability in total signal within and between runs.

### 2.3 Statistical analysis

Descriptive statistics were used to summarize patient demographics and clinical characteristics. Comparisons between survivors and non‐survivors were summarized using Pearson's chi‐squared test of independence or Welch's *t*‐test. The Bioconductor package *limma* using the robust *limma*‐trend method (Law et al., 2014; Phipson et al., 2016) tested differences in protein expression values from Day 1 to Day 7 and further evaluated the association between proteomic expression values and SOFA scores at Day 1, Day 7, and the changes between Days 1 and 7. The *limma* method fits linear models to the expression data for each protein using a moderated *t*‐test, which is an empirical Bayes procedure where the observed protein sample variance is shrunk towards the common prior value. Repeated measurements on subjects were handled using the *duplicateCorrelation* function in *limma*. The normalized values from the Somalogic adaptive normalization procedure were converted to log2 counts per million prior to modeling. Effect size estimates from limma analyses were summarized as a log2 fold‐change, which represents the estimated relative change in protein abundance for an interquartile range (IQR) change in SOFA score. Ordinary least squares (OLS) were used to estimate the relationships between the change in SOFA score from day 1 to day 7 and change in protein abundance from day 1 to day 7. Effect size estimates from OLS represent the estimated difference in SOFA score (day 7 – day 1) for a one‐unit log2 fold‐change in protein abundance (day 7/day 1). All models in *limma* and OLS analyses were adjusted for subject age, sex, and BMI. False discovery rate (FDR) adjusted p‐values were used to control the proportion of false positives among the set of proteins declared differentially abundant at a threshold of less than 0.1 and used to determine protein lists for plotting and pathway analyses. Heatmaps, volcano plots, and scatterplots explored significant results visually. Analyses were performed using R (Team RDC, 2024) (version 4.3.3) with the limma (Ritchie et al., 2015) (version 3.58.1), ggplot2 (Wickham, 2016) (version 3.5.1), and pheatmap (Kolde, 2019) (version 1.0.12) add‐on packages.

### 2.4 Ingenuity pathway analysis

We utilized Ingenuity Pathway Analysis (IPA v24.0, Qiagen) software to analyze the resultant protein abundance data to identify canonical pathways common to the differentially abundant proteins (DAPs) in each comparison. Analysis results were uploaded to IPA along with their corresponding log2FC and adjusted *p* values. Pathways were filtered for significance at a −log10 *p* value of 2 (using a B‐H Multiple Testing Correction *p*‐value), which corresponds to a false discovery rate (FDR) of 0.01. An absolute z‐score of 0.5 was used as a cut‐off for pathway directionalities. Volcano plots were generated with IPA software.

## 3 RESULTS

### 3.1 Subject characteristics

To characterize dynamic circulating protein changes correlated with disease severity across time in critically ill patients with COVID‐19 ARDS, we identified a patient cohort from the Ohio State University Intensive Care Unit Registry (BuckICU), an existing, single‐center biorepository containing longitudinal patient biospecimens and clinical data. Our study was designed to identify differences in plasma proteomes between survivors and non‐survivors. We identified eligible participants with COVID‐19 ARDS and available day 1 and day 7 plasma samples. To establish our analysis cohort, subjects were matched by age, male sex, white race, and body mass index (BMI) to establish our analysis cohort. With this primary analysis, no proteins demonstrated significant differential abundance between survivors and non‐survivors at day 1, day 7, or changes across time, possibly reflecting inadequate power given the limited sample size. For secondary analyses, we compared protein expression related to indices of disease severity, specifically P/F ratio (partial pressure of oxygen in arterial blood compared to the fraction of oxygen the patient is receiving) and SOFA scores. The plasma proteome did not show a significant correlation with P/F ratios in our study. We did identify a significant correlation between protein abundance and SOFA scores. Subject characteristics of the analyzed cohort are shown in Table 1. All patients met ARDS criteria during ICU admission, required mechanical ventilation with low tidal volume ventilation, and received standard ICU supportive care. P/F ratios and SOFA scores trended towards improvement from Day 1 to Day 7 with heterogeneous patient clinical courses. All patients met sepsis criteria, per the Sepsis‐3 definition. These patients received treatment per current sepsis care guidelines (Dellinger et al., 2008, 2013), including source control, early antibiotics, and volume resuscitation. All COVID‐19 subjects tested positive for SARS‐CoV‐2 by upper respiratory tract nucleic acid amplification test, and all subjects were unvaccinated against SARS‐CoV‐2. Patients received treatments for COVID‐19, per established guidelines at the time of hospital admission.

**TABLE 1:** Subject characteristics.

Characteristics	
*N*	32
Age	59.10 (15.08)
Male sex	18 (56.3%)
White race	27 (84.4%)
BMI	37.28 (10.42)
In hospital mortality	18 (56.3%)
Smoking	
Current	1 (3.1%)
Former	12 (37.5%)
Never	18 (56.3%)
Unknown	1 (3.1%)
P/F Ratio	
Day 1	103.67 (27.92)
Day 7	126.46 (49.23)
SOFA Score	
Day 1	9.69 (3.00)
Day 7	8.53 (4.03)
Bacteremia	
Negative	20 (62.5%)
Not available	9 (28.1%)
Positive	3 (9.4%)
Day 1 WBC	13.74 (7.22)
Day 1 Vasopressors	15 (46.9%)
Day 1 Mechanical Ventilation	32 (100%)
Day 1 Sepsis Criteria	32 (100%)
Remdesivir	25 (78.1%)
Convalescent Plasma	10 (31.3%)
Steroids	26 (81.3%)

*Note*: Values shown as mean (SD) or percentage of subjects.

### 3.2 Plasma proteomics identifies known processes of host defense during SARS‐CoV‐2 infection

**FIGURE 2:**
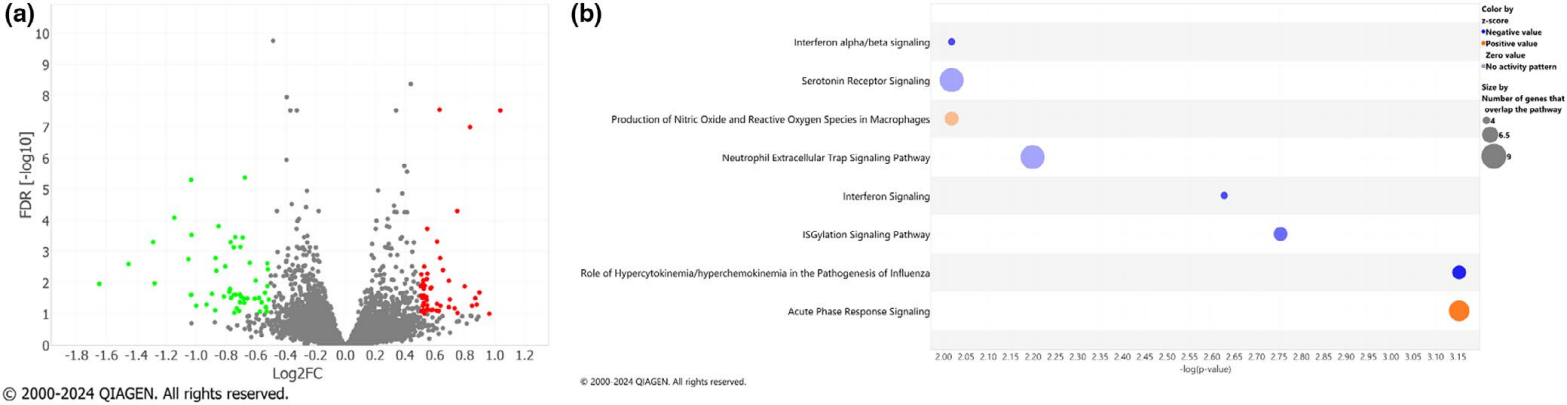
Differentially Abundant Proteins (DAPs) between days 1 and 7 for all analyzed samples. (a) Volcano plot of DAPS on Day 7 compared to Day 1. Red dots indicate increased protein abundance (positive fold change) on Day 7 compared to Day 1. Green dots indicate decreased protein abundance. Significance was defined as an adjusted *p*‐value (FDR) <0.1 and log2FC absolute value >0.5. The upregulated DAPS are represented by red dots and the downregulated proteins are in green. (b) Ingenuity pathway analysis (IPA) of the canonical pathways identified by the longitudinal analysis. The size of bubbles correlates the number of genes overlapping the pathway and the color corresponds to the directionality of the pathway, where the orange represents a positive z‐score (increased on Day 7 compared to Day 1) and the blue represents downregulation of a pathway. Here, −log (*p*‐value) >2.0 (equivalent to *p*‐value <0.01) and z‐sore >0.5 were used to filter significant pathways.

We used an unbiased, aptamer‐based array (SomaScan 7 k platform, SomaLogic, Inc.) to determine the protein abundance of over 7000 proteins in patient plasma from 32 patients at both days 1 and 7 of ICU admission. We first characterized the dynamic changes of the circulating plasma proteome between days 1 and 7 by differential abundance analysis, modeled by *limma* with a false discovery rate (FDR) of 0.1 and a log2FC absolute value of 0.5. We identified 119 differentially abundant proteins (DAPs) between time points, with 62 upregulated and 57 downregulated DAPs at day 7 compared to day 1 (Figure 2a). The top 20 proteins demonstrating the most significance between days 1 and 7 are shown in Table 2. Among the top 20 DAPs, proteasome 20S subunit alpha 4 (PSMA4), TNF receptor superfamily member 11a (TNFRSF11A), carbonic anhydrase 12 (CA12), and ABL proto‐oncogene 2, non‐receptor tyrosine kinase (ABL2) increased across time. Tubulin polymerization promoting protein family member 2 (TPPP2), protein tyrosine phosphatase non‐receptor type 7 (PTPN7), decapping mRNA 1B (DCP1B), and KIT proto‐oncogene, receptor tyrosine kinase (KIT) decreased between days 1 and 7. To better understand common biological pathways modulated during the COVID‐19 ARDS disease course, we used Ingenuity Pathway Analysis (IPA, Qiagen) to identify canonical signaling pathways implicated by our DAP analysis. Between days 1 and 7, we observed significantly increased activation of acute phase response signaling and production of nitric oxide and reactive oxygen species in macrophages, while ISGylation, interferon, and neutrophil extracellular trap signaling were downregulated (Figure 2b). These findings recapitulate many known processes during the host response to severe SARS‐CoV‐2 infection, specifically early interferon signaling and variation in innate immune responses in a mixed population of patients with severe COVID‐19.

**TABLE 2:** Top 20 most significant differentially abundant proteins on Day 7 compared to Day 1.

Symbol	Entrez gene name	Log2FC	Adjusted *p* value
PITX3	Paired like homeodomain 3	−0.484	1.72E‐10
PSMA4	Proteasome 20S subunit alpha 4	0.438	4.20E‐09
KIF3B	Kinesin family member 3B	−0.393	1.10E‐08
ABL2	ABL proto‐oncogene 2, non‐receptor tyrosine kinase	0.631	2.80E‐08
PTPN7	Protein tyrosine phosphatase non‐receptor type 7	−0.325	2.94E‐08
CA12	Carbonic anhydrase 12	0.339	2.94E‐08
SNN	Stannin	1.037	2.94E‐08
DCP1B	Decapping mRNA 1B	−0.369	2.94E‐08
FCN1	Ficolin 1	0.835	1.01E‐07
ZBTB10	Zinc finger and BTB domain containing 10	−0.395	1.13E‐06
RABIF	RAB interacting factor	0.394	1.75E‐06
EMID1	EMI domain containing 1	0.413	2.74E‐06
RFX5	Regulatory factor X5	−0.673	4.19E‐06
TPPP2	Tubulin polymerization promoting protein family member 2	−1.033	4.95E‐06
ERBB4	erb‐b2 receptor tyrosine kinase 4	0.219	1.10E‐05
ARMC8	Armadillo repeat containing 8	−0.258	1.13E‐05
TNFRSF11A	TNF receptor superfamily member 11a	0.381	1.36E‐05
AKAP7	A‐kinase anchoring protein 7	−0.36	3.02E‐05
RBKS	Ribokinase	0.326	3.32E‐05
DNAJB14	DnaJ heat shock protein family (Hsp40) member B14	−0.263	3.74E‐05

*Note*: Values are log2 fold change (Log2FC) and p‐value adjusted for multiple comparisons of day 7 protein abundance compared to day 1 by limma method. Positive Log2FC indicates protein abundance was higher on day 7 compared to day 1, while negative Log2FC indicates decreased protein abundance on day 7 compared to day 1.

### 3.3 Plasma proteomics reveals protein signatures correlated with organ dysfunction scoring during COVID‐19 ARDS

After observing changes in the circulating proteome of patients with COVID‐19 ARDS across time, we hypothesized that protein abundance may correlate with disease severity. Here, we used the sequential organ failure assessment score (SOFA score) as a measure of disease severity at each time point. Critical illness is dynamic and does not adhere to a strict temporal progression. Comparing protein abundance among categorical groups defined by mortality is limited to 2 categories (survivors versus non‐survivors) and is confounded by time to mortality. SOFA scores are timely, reflecting disease severity at the time of sampling, and can be analyzed as a continuous variable. Accounting for heterogeneity in our cohort of critically ill patients, we used interquartile ranges (IQR) to determine DAPs that correlate with SOFA scores. The estimated log2 fold change (log2FC) is interpreted as the change in protein abundance for SOFA scores observed in the first quartile to the observed third quartile values (IQR unit basis). As an example, a log2FC of 1 indicates that the protein abundance is expected to double for a SOFA score in the third quartile compared to the first quartile, and a log2FC of −1 indicates a protein abundance of 0.5 for SOFA scores in the third quartile compared to the first quartile. We used an FDR of <0.1 to determine significance and correlated protein abundance with SOFA scores for each subject at day 1 and 7 of ICU admission.

**FIGURE 3:**
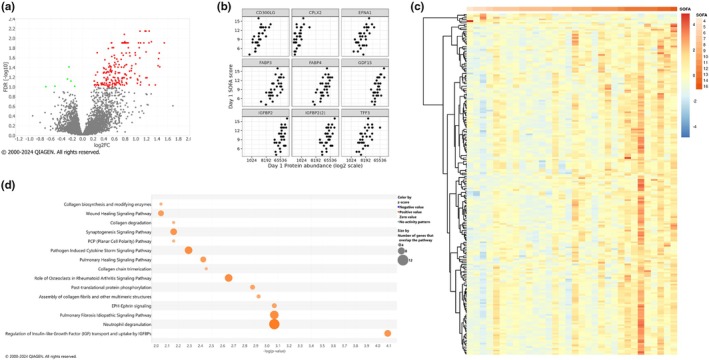
Day 1 differentially abundant proteins by SOFA scores. (a) Volcano plot of significant DAPS by SOFA scores on Day 1. Significance was defined as an adjusted *p*‐value (FDR) <0.1. DAPS positively correlated with SOFA score are red; negative correlations are green. (b) Scatter plots of the top 9 most significant DAPs, ordered alphabetically, plotting Day 1 array signaling intensity relative to protein abundance (*x*‐axis) against Day 1 SOFA scores (*y*‐axis). (c) Heat map illustrating the significant DAPS (FDR <0.1) with columns ordered by SOFA score. Blue to red color scale represents log2fold change. (d) Ingenuity pathway analysis (IPA) of canonical pathways for Day 1. The size of bubbles correlates the number of genes overlapping the pathway and the color corresponds to the directionality of the pathway, where the orange represents a positive z‐score (upregulated) and the blue represents downregulation of a pathway. Here, −log (*p*‐value) >2.0 (equivalent to *p*‐value <0.01) and a z‐sore >0.5 were used to filter significant pathways.

At day 1, we identified 184 DAPs with 178 proteins positively correlated with SOFA scores (increased protein abundance with increased SOFA score) and 6 negatively correlated with SOFA scores (Figure 3a,c). For 9 proteins determined to be highly statistically significant, we plotted protein abundance against SOFA scores and observed positive correlations (Figure 3b). The 20 DAPs with the most significant association (lowest FDR) with SOFA score on day 1 all demonstrated a positive association with SOFA score, as shown in Table 3. Included among these are multiple ephrin ligands (A1, A2, and A5), immune response‐related proteins, such as trefoil factor 3 (TFF3), follistatin like 3 (FSTL3), and delta like canonical Notch ligand 1 (DLL1), and lipid metabolism related proteins, such as fatty acid binding protein 3 (FABP3), fatty acid binding protein 4 (FABP4), and prostaglandin D2 synthase (PTGDS). Again, we used Ingenuity Pathway Analysis (IPA) and identified activation of canonical pathways including regulation of insulin‐like growth factor (IGF) transport and uptake by IGFBPs, neutrophil degranulation, EPH‐ephrin signaling, pathogen induced cytokine storm signaling, and pulmonary healing signaling pathways. These findings suggest that plasma proteomics can identify biomarkers early in the course of severe respiratory failure that correlate with disease severity in COVID‐19 ARDS.

**TABLE 3:** Top 20 most significant differentially abundant proteins by SOFA score on Day 1.

Symbol	Entrez gene name	Log2FC	Adjusted *p* value
TFF3	Trefoil factor 3	1.19	7.13E‐03
CD300LG	CD300 molecule like family member g	1.17	7.13E‐03
EFNA1	Ephrin A1	1.25	7.13E‐03
FABP4	Fatty acid binding protein 4	1.08	7.15E‐03
CPLX2	Complexin 2	0.78	8.32E‐03
FABP3	Fatty acid binding protein 3	1.29	1.18E‐02
GDF15	Growth differentiation factor 15	0.79	1.18E‐02
EFNA5	Ephrin A5	0.74	1.25E‐02
DLL1	Delta like canonical Notch ligand 1	0.54	1.25E‐02
SELENOM	Selenoprotein M	0.54	1.25E‐02
DSC2	Desmocollin 2	0.83	1.25E‐02
EFNA2	Ephrin A2	0.78	1.25E‐02
THY1	Thy‐1 cell surface antigen	0.86	1.25E‐02
PTGDS	Prostaglandin D2 synthase	0.77	1.25E‐02
CFD	Complement factor D	0.76	1.25E‐02
FSTL3	Follistatin like 3	0.81	1.25E‐02
HSPB6	Heat shock protein family B (small) member 6	1.05	1.25E‐02
IGFBP2	Insulin like growth factor binding protein 2	1.15	1.25E‐02
RBP7	Retinol binding protein 7	0.80	1.25E‐02
PDLIM3	PDZ and LIM domain 3	1.54	1.25E‐02

*Note*: Values are log2 fold change (Log2FC) of day 1 protein abundance correlated to interquartile SOFA score on day 1 by limma method. A log2FC of 1 indicates that the protein abundance is expected to double for a SOFA score in the third quartile compared to the first quartile.

**FIGURE 4:**
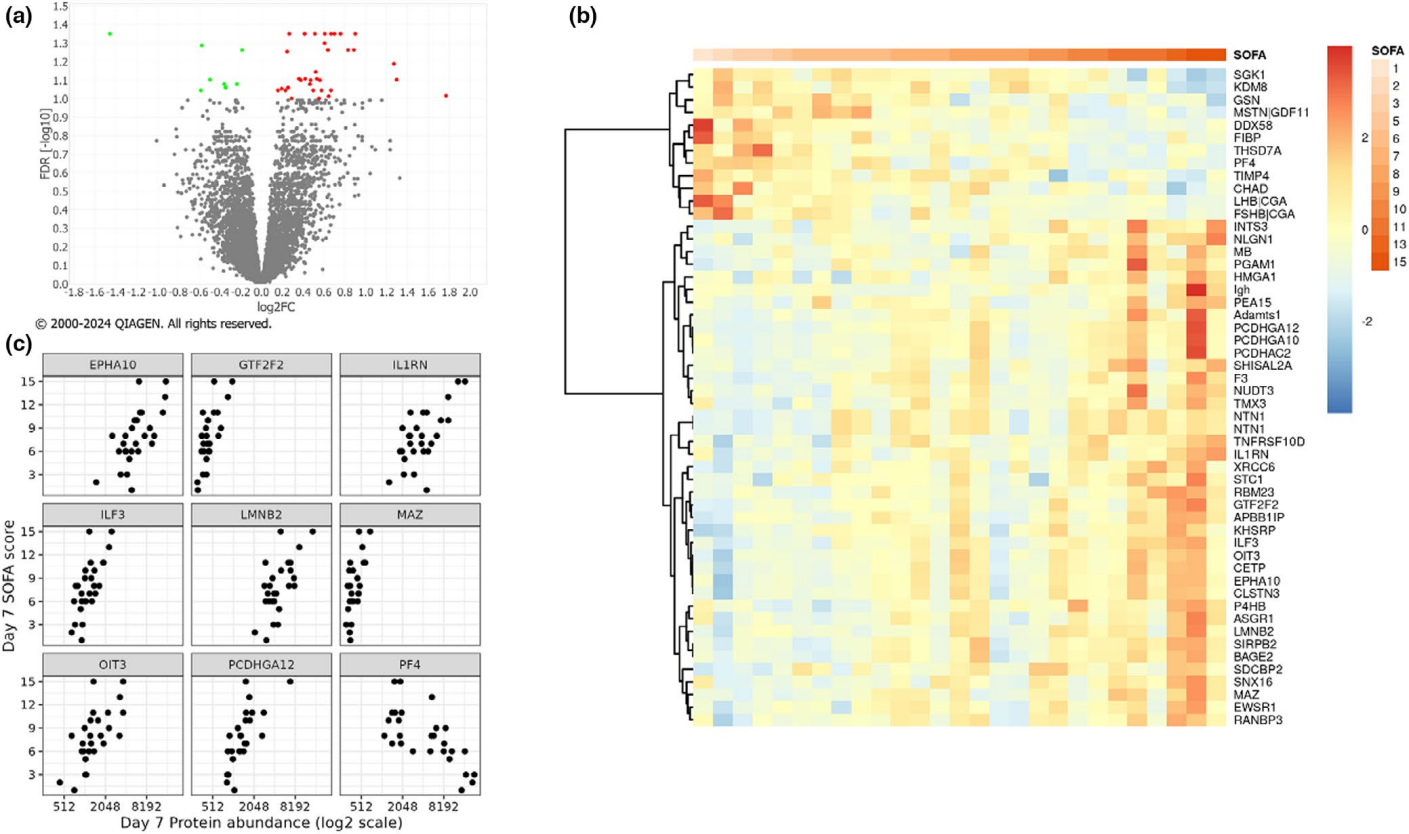
Day 7 differentially abundant proteins by SOFA scores. (a) Volcano plot of significant DAPS by SOFA scores on Day 7. Significance was defined as an adjusted *p*‐value (FDR) <0.1. DAPS positively correlated with SOFA score are red; negative correlations are green. (b) Heat map illustrating the significant DAPS (FDR <0.1) with columns ordered by SOFA score. Blue to red color scale represents log2fold change. (c) Scatter plots of the top 9 most significant DAPs, ordered alphabetically, plotting Day 7 protein abundance (x‐axis) against Day 7 SOFA scores (*y*‐axis).

At day 7, we identified 46 DAPs with 37 proteins positively correlated with SOFA scores and 9 negatively correlated with SOFA scores (Figure 4a,b). Notable proteins among the top 20 DAPS include interleukin 1 receptor antagonist (IL1RN), multiple transcription factors, such as general transcription factor IIF subunit 2 (GTF2F2), interleukin enhancer binding factor 3 (ILF3), and MYC associated zinc finger protein (MAZ), and numerous cell adhesion proteins, including protocadherin gamma subfamily A, 12 (PCDHGA12), protocadherin gamma subfamily A, 10 (PCDHGA10), and protocadherin alpha subfamily C, 2 (PCDHAC2). Unlike day 1, no ephrin ligands were significantly associated with SOFA at day 7. However, ephrin receptor A10 (EPHA10) had a significant positive correlation with day 7 SOFA scores (Figure 4c, Table 4). We next pursued IPA pathway analysis of DAPs at day 7. However, the identified pathways did not meet the threshold for statistical significance.

**TABLE 4:** Top 20 most significant differentially abundant proteins by SOFA score on Day 7.

Symbol	Entrez gene name	Log2FC	Adjusted P.Value
GTF2F2	General transcription factor IIF subunit 2	0.41	4.47E‐02
PCDHGA12	Protocadherin gamma subfamily A, 12	0.67	4.47E‐02
SIRPB2	Signal regulatory protein beta 2	0.42	4.47E‐02
PF4	Platelet factor 4	−1.45	4.47E‐02
LMNB2	Lamin B2	0.61	4.47E‐02
OIT3	Oncoprotein induced transcript 3	0.76	4.47E‐02
CLSTN3	Calsyntenin 3	0.70	4.47E‐02
ILF3	Interleukin enhancer binding factor 3	0.51	4.47E‐02
IL1RN	Interleukin 1 receptor antagonist	0.90	4.47E‐02
MAZ	MYC associated zinc finger protein	0.27	4.47E‐02
EPHA10	EPH receptor A10	0.76	4.47E‐02
CETP	Cholesteryl ester transfer protein	0.61	5.03E‐02
CHAD	Chondroadherin	−0.57	5.16E‐02
PCDHGA10	Protocadherin gamma subfamily A, 10	0.83	5.46E‐02
BAGE2	BAGE family member 2, pseudogene	0.64	5.46E‐02
SGK1	Serum/glucocorticoid regulated kinase 1	−0.19	5.46E‐02
PCDHAC2	Protocadherin alpha subfamily C, 2	0.64	5.46E‐02
SHISAL2A	Shisa like 2A	0.89	5.46E‐02
NLGN1	Neuroligin 1	0.25	5.58E‐02
MB	Myoglobin	1.27	6.50E‐02

*Note*: Values are log2 fold change (Log2FC) of day 7 protein abundance correlated to interquartile SOFA score on day 7 by limma method. A log2FC of 1 indicates that the protein abundance is expected to double for a SOFA score in the third quartile compared to the first quartile.

### 3.4 Longitudinal plasma proteomics correlates with dynamic clinical course

**FIGURE 5:**
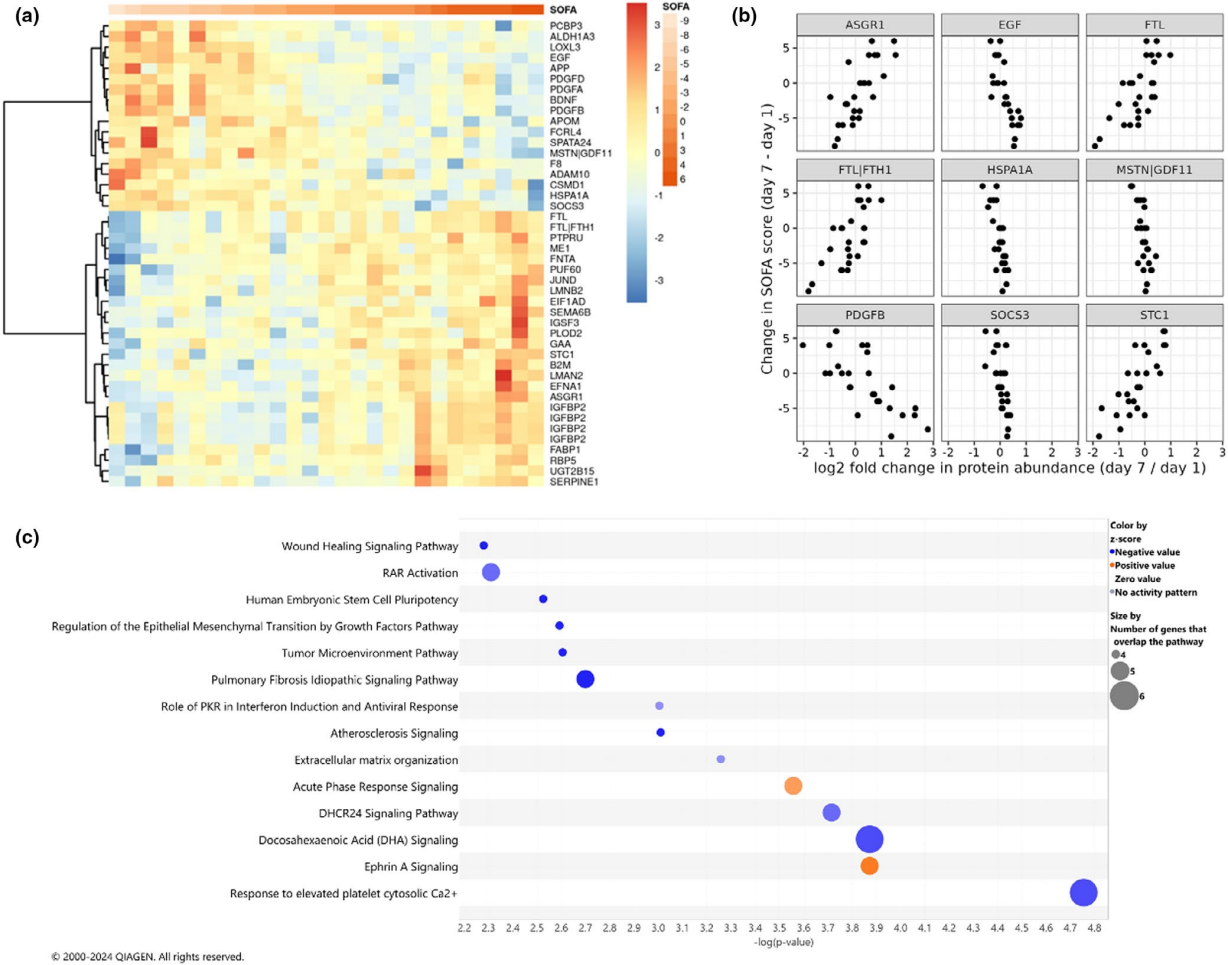
Changes in protein abundance correlate to changes in SOFA scores between Days 1 and 7. (a) Heat map of the significant DAPS (FDR <0.1) when modeling change in protein abundance as an explanatory variable for change in SOFA scores between Days 1 and 7 with columns ordered by difference in SOFA scores over time (Days 7 SOFA score minus Day 1 SOFA score). Blue to red color scale represents log2fold change. (b) Scatter plots of the top 9 most significant DAPs, ordered alphabetically, plotting log2 fold change in protein abundance (*x*‐axis) against change in SOFA score (*y*‐axis). (c) Ingenuity pathway analysis (IPA) of canonical pathways for Day 1. The size of bubbles correlates the number of genes overlapping the pathway and the color corresponds to the directionality of the pathway, where the orange represents a positive z‐score (upregulated) and the blue represents downregulation of a pathway. Here, −log (*p*‐value) >2.0 (equivalent to *p*‐value <0.01) and a z‐sore >0.5 were used to filter significant pathways.

Patient heterogeneity and dynamic kinetics of disease complicate molecular profiling studies in critical illness. We next hypothesized that changes in plasma proteome correlate with changes in SOFA scores across the two measured time points, days 1 and 7. Here, we calculated the log2 fold change in protein abundance from day 1 to day 7 and used that as an explanatory variable for a linear model with the difference in SOFA score between days 1 and 7 as the outcome. This approach calculates an estimated difference in SOFA score between days 1 and 7 based on a 1 unit change in log2FC with a positive score for a positive association and a negative score for a negative association. This analysis identified differentially abundant signals from 44 aptamers corresponding to 40 distinct proteins, specifically 22 proteins with a change in protein abundance positively correlated with a change in SOFA scores (protein increases and SOFA increases) and 18 negatively correlated with SOFA scores (Figure 5a). Proteins that increased with correlated SOFA increases included stanniocalcin 1 (STC1), ferritin light chain (FTL), ephrin A1 (EFNA1), and insulin‐like growth factor binding protein 2 (IGFBP2). Proteins with a negative correlation with SOFA included heat shock protein family A (Hsp70) member 1A (HSPA1A/HSPA1B), epidermal growth factor (EGF), platelet‐derived growth factor subunit B (PDGFB), and suppressor of cytokine signaling 3 (SOCS3) (Figure 5b, Table 5). IPA analysis revealed two pathways with a positive correlation to SOFA scores: ephrin A signaling and acute phase response signaling. Notable pathways negatively associated with SOFA score change were response to elevated platelet cytosolic Ca2+ signaling, docosahexaenoic acid (DHA) signaling, 24‐dehydrocholesterol reductase (DHCR24) signaling, pulmonary fibrosis idiopathic signaling, and wound healing signaling.

**TABLE 5:** Top 20 most significant proteins using change in protein abundance to estimate change in SOFA score between days 1 and 7.

Symbol	Entrez gene name	Estimate	Adjusted P.Value
STC1	Stanniocalcin 1	4.89	1.31E‐02
HSPA1A/HSPA1B	Heat shock protein family A (Hsp70) member 1A	−14.78	1.31E‐02
EGF	Epidermal growth factor	−9.31	1.31E‐02
ASGR1	Asialoglycoprotein receptor 1	5.03	1.31E‐02
PDGFB	Platelet derived growth factor subunit B	−2.78	1.84E‐02
FTL	Ferritin light chain	4.86	2.31E‐02
SOCS3	Suppressor of cytokine signaling 3	−13.60	2.64E‐02
EFNA1	Ephrin A1	4.88	3.03E‐02
PTPRU	Protein tyrosine phosphatase receptor type U	4.09	3.03E‐02
APP	Amyloid beta precursor protein	−6.77	3.42E‐02
IGFBP2	Insulin like growth factor binding protein 2	3.55	4.56E‐02
FNTA	Farnesyltransferase, CAAX box, alpha	7.39	4.59E‐02
LMNB2	Lamin B2	4.16	4.59E‐02
ADAM10	ADAM metallopeptidase domain 10	−29.34	4.59E‐02
ALDH1A3	Aldehyde dehydrogenase 1 family member A3	−9.80	4.74E‐02
PDGFA	Platelet derived growth factor subunit A	−5.41	4.78E‐02
LMAN2	Lectin, mannose binding 2	6.35	4.92E‐02
PUF60	Poly(U) binding splicing factor 60	5.35	6.16E‐02
FABP1	Fatty acid binding protein 1	2.88	6.16E‐02
ME1	Malic enzyme 1	3.41	6.16E‐02

*Note*: Estimate represents the change in SOFA score for a 1 unit change in log2FC protein abundance between days 1 and 7 by limma method.

## 4 DISCUSSION

Critical illness syndromes, including ARDS and sepsis, are heterogeneous and dynamic. Targeted therapies are lacking. To better understand the dynamic mechanisms of disease, we performed high throughput plasma proteomics at two time points and correlated protein abundance with disease severity, measured by SOFA scores in this cohort of critically ill patients with COVID‐19. At day 1 of ICU admission, we observed multiple pathways related to innate immune signaling, repair mechanisms, and ephrin signaling were upregulated in patients with higher SOFA scores. In a longitudinal analysis correlating differential protein abundance between days 1 and 7 with changes in SOFA score across time, we observed activated ephrin A and acute phase response signaling correlating with increased SOFA scores. Conversely, activation of lipid‐related metabolism pathways and wound healing correlated with improvement in SOFA scores. Taken together, these findings suggest an important interplay between persistent inflammation and repair processes that function during COVID‐19 ARDS and suggest targets for further mechanistic investigation.

SARS‐CoV2 emerged in 2019 and became the most common risk factor for ARDS during the pandemic (Wiersinga et al., 2020; Yang et al., 2020). Some reports have suggested that COVID‐19 ARDS is different from prior observed cases of ARDS (Bickler et al., 2021; Gattinoni et al., 2020; Lu et al., 2022). However, ARDS is known to be heterogeneous in cause, disease course, and underlying mechanisms of injury and repair. Study of ARDS subgroups provides an opportunity to identify both specific mechanisms of disease and uncover unifying features driving ARDS disease severity and recovery. In this discovery cohort of patients with COVID‐19 ARDS, we first explored dynamic changes in circulating proteins between days 1 and 7, irrespective of disease severity or outcome. We observed evidence of persistent activation of innate immune responses and downregulation of interferon signaling, consistent with prior reports in severe COVID‐19 (Diao et al., 2020; Guan et al., 2020; Hadjadj et al., 2020; Janssen et al., 2021; Lee et al., 2021; Mohammed et al., 2022; Qin et al., 2020; Wu et al., 2022). Several studies in COVID‐19 patients have examined the plasma proteome, either at a single (Babačić et al., 2023; Hou et al., 2020; Janssen et al., 2021; Patel et al., 2021; Sullivan et al., 2021; Völkel et al., 2023; Wilson et al., 2020) or multiple time points (Duijvelaar et al., 2024; Ebihara et al., 2022; Filbin et al., 2021; Haljasmägi et al., 2020; Iosef et al., 2023; Lee et al., 2021; Mohammed et al., 2022; Wu et al., 2022; Zhong et al., 2021). Comparator groups of COVID‐19 severity were pre‐defined by level of care (hospitalized, ICU), respiratory support (ventilated vs. non‐ventilated), or mortality. Longitudinal studies reported comparisons between groups at each individual time point. Our study aimed to extend beyond these findings. First, use of SOFA scores to define severity enables a “real‐time” snapshot of disease severity. Second, comparison of changes in proteome and SOFA across time may account for individual patient baselines, where the extent of change rather than an absolute measure is important, to uncover dynamic processes driving divergent disease course. Our longitudinal approach aimed to identify active host response mechanisms and further, to characterize interplay between response pathways that may determine COVID‐19 ARDS clinical course.

Through multiple analyses, we examined the correlation between organ dysfunction and protein abundance both at each time point (Day 1 protein with Day 1 SOFA; Day 7 protein with Day 7 SOFA) and across time (change in protein versus change in SOFA). Echoing findings from prior COVID‐19 studies that suggest heightened innate immune activation as a driver of disease severity (Abers et al., 2021; Janssen et al., 2021; Lee et al., 2021; Leisman et al., 2022; Mohammed et al., 2022; Wu et al., 2022; Wu, Chen, et al., 2020), we identified a correlation between activated innate immune response pathways and organ dysfunction at Day 1, including neutrophil degranulation and pathogen‐induced cytokine storm signaling. In our longitudinal analysis, increased acute phase response signaling correlated with increased SOFA scores. These findings suggest that persistent innate immune activation drives organ injury and dysfunction. Notably, pulmonary fibrosis idiopathic signaling and wound healing signaling pathways showed a positive relationship with SOFA score at day 1, but in our longitudinal analysis, activation of these pathways over time correlated with improved organ dysfunction scores. Collectively, these findings suggest that early in the disease course, lung fibrotic responses, wound healing, and collagen pathways, which may represent lung repair processes, are simultaneously upregulated with innate immune responses. However, later in the disease course, failure of repair mechanisms and persistent inflammation predict increased organ dysfunction and disease severity. Consistent with our findings, prior investigations of histopathologic findings in COVID‐19 have observed lung fibrosis and fibroblast proliferation (Gál et al., 2022; Valdebenito et al., 2021). Extracellular matrix (ECM) is known to have a key role in lung homeostasis and injury response (Huang et al., 2023; Zhou et al., 2018), and SARS‐CoV‐2 modifies the lung ECM with the release of ECM proteins into the circulating blood (D'Agnillo et al., 2021; Rauti et al., 2021; Zhou et al., 2018). We observed multiple upregulated collagen proteins associated with SOFA scores at Day 1. Other COVID‐19 proteomic studies demonstrated modulation of ECM proteins, including collagens, in critically ill COVID‐19 patients (Duijvelaar et al., 2024; Leng et al., 2020).

One notable observation common among all three analyses was the positive association between ephrin proteins, ephrin signaling pathways, and SOFA score. Ephrins have an important role in inflammatory (Dixit et al., 1990; Ivanov & Romanovsky, 2006) and immune response to infection (Darling & Lamb, 2019; Funk & Orr, 2013), including CD8 and CD4 T cell migration (Saintigny et al., 2012), and further have been shown to regulate endothelial (Chan & Sukhatme, 2009) and lung vascular permeability during injury (Larson et al., 2008). Previous studies demonstrated a positive association between COVID‐19 severity and ephrin signaling in blood (ephrin ligand A1) (Mendoza et al., 2021), saliva (ephrin ligands A1 and B2) (Egal et al., 2022), but a negative correlation in urine (Ephrin receptors B2,3, and 6) (Bi et al., 2022). Our study, in agreement with these prior reports, implicates ephrin proteins in the pathobiology of lung injury and supports further investigation of ephrins and ephrin signaling as potential biomarkers or therapeutic targets for COVID‐19.

Our analyses also identified multiple pathways that suggest altered metabolic regulation during COVID‐19. The most significant pathway associated with SOFA score at Day 1 was regulation of insulin growth factor transport and uptake by IGFBPs, represented by upregulation of insulin‐like growth factor (IGF) binding proteins 1,2,4 and 6 (IGFBP). IGFBPs decrease the free IGF circulating levels (Lang et al., 2003; Muzumdar et al., 2006; Russell‐Jones et al., 1995). Previous COVID‐19 studies have shown IGFBPs to be associated with more severe disease (Iosef et al., 2023; Mester et al., 2024; Mohammed et al., 2022), while IGF levels are higher in mild COVID‐19 than in the severe form (Hazrati et al., 2022; Iosef et al., 2023). Our longitudinal analysis identified lipid metabolism‐related pathways among the most significant pathways, including Docosahexaenoic ACID (DHA, a member of the Omega‐3 family of essential fatty acids) signaling, DHCR24 Signaling (cholesterol synthesis), and atherosclerotic signaling pathways. Previous studies have shown dysregulated lipid profiles during COVID‐19 (Jin et al., 2021; Mahat et al., 2021; Wu, Shu, et al., 2020). Moreover, COVID‐19 ARDS is associated with proinflammatory lipid mediators including prostaglandin D2 (PGD2) (Archambault et al., 2021), which produces IL‐13 and is associated with respiratory failure (Ogletree et al., 2022). These findings highlight the systemic nature of COVID‐19 and suggest other putative mechanisms of disease severity.

Our study used longitudinal circulating proteomics to identify putative drivers of disease during COVID‐19 ARDS. This approach is generalizable to larger COVID‐19 and ARDS cohorts but is limited by several factors. First, our study was limited to a single center, which may not be representative of the larger population. Subjects were enrolled from May 2020 to June 2021, prior to widespread vaccination and population exposure to SARS‐CoV‐2. We identified patients using ARDS and COVID‐19 as inclusion criteria. However, all patients met criteria for sepsis by the Sepsis‐3 guidelines (Guarino et al., 2023; Singer et al., 2016), which is indicative of severe COVID‐19 as a systemic disease. We did not attempt to attribute our findings specifically to ARDS, sepsis, or both as independent groups. Among critically ill patients, often overlapping syndrome‐based paradigms (ARDS, sepsis, severe pneumonia) currently limit such analysis approaches (Maslove et al., 2022; Wang et al., 2024). This study analyzed the circulating plasma proteome, which may reflect systemic responses as opposed to the lung‐specific during COVID‐19 ARDS. We used an aptamer‐based protein array to quantify protein abundance. While the SOMAScan method is a validated tool that allows for high throughput, scalable, and consistent measurement of protein abundance in complex biologic fluids, the SOMAScan platform is vulnerable to several sources of potential error. SOMAScan aptamers are multiplexed (over 7000 proteins) may exhibit cross‐reactivity or non‐specific binding with non‐target proteins, and protein sequence variation may impact aptamer binding efficiency (Joshi & Mayr, 2018). Finally, our study was designed as a discovery cohort. We do not present a validation cohort and did not pursue top result validation with an orthogonal method.

Due to heterogeneity among critically ill patients, targeted treatment options for patients with ARDS and sepsis remain limited. COVID‐19 is no exception. Studies characterizing molecular drivers of disease are paramount to progress towards personalized medicine in the ICU. Our study identified differentially abundant proteins that correlate with dynamic disease severity. We identified pathways of increased ephrin signaling and acute phase responses and downregulated lipid metabolism‐related proteins, pulmonary fibrotic signaling, and wound healing pathways that correlated with increased SOFA scores, as an index for organ dysfunction. Further, our results identified multiple individual proteins including ephrins, IGFBPs, and DLL1, previously identified as biomarkers in COVID‐19 (Baindara et al., 2022; Breikaa & Lilly, 2021; Egal et al., 2022; Mendoza et al., 2021; Mester et al., 2024). Our longitudinal design helps to untangle the temporal overlap of these pathways and suggests that short interval longitudinal sampling during acute illness can better characterize mechanisms of injury and repair during critical illness. While specific proteins and pathways identified in this discovery cohort require further validation, our approach is generalizable to larger studies and builds towards individualized medicine for critically ill patients.

## AUTHOR CONTRIBUTIONS

ME: Conceived and designed research, analyzed data, interpreted results of experiments, prepared figures, drafted manuscript, edited and revised manuscript, approved final version of manuscript. BK: Performed experiments, analyzed data, interpreted results of experiments, prepared figures, edited and revised manuscript, approved final version of manuscript. DF: Performed experiments, edited and revised manuscript, approved final version of manuscript. JDL: Conceived and designed research, edited and revised manuscript, approved final version of manuscript. JAE: Conceived and designed research, interpreted results of experiments, edited and revised manuscript, approved final version of manuscript. JCH: Conceived and designed research, analyzed data, interpreted results of experiments, edited and revised manuscript, approved final version of manuscript. RKM: Conceived and designed research, interpreted results of experiments, edited and revised manuscript, approved final version of manuscript. GB: Conceived and designed research, analyzed data, interpreted results of experiments, edited and revised manuscript, approved final version of manuscript. JSB: Conceived and designed research, performed experiments, analyzed data, interpreted results of experiments, prepared figures, drafted manuscript, edited and revised manuscript, approved final version of manuscript.

## FUNDING INFORMATION

This project was supported by NIH K08HL169725 awarded to J.S.B., R01HL142767 awarded to J.A.E., R01HL141195 awarded to J.C.H., P01HL114453, R01HL097376, R01HL081784, and R01HL096376 awarded to R.K.M. This publication was supported, in part, by The Ohio State University Clinical and Translational Science Institute (CTSI) and the National Center for Advancing Translational Sciences of the National Institutes of Health under Grant Number UM1TR004548. The content is solely the responsibility of the authors and does not necessarily represent the official views of the National Institutes of Health. This project was further supported by the Ohio State University Office of Research 2020 COVID‐19 Seed Funding Program and the Department of Internal Medicine 2021 Junior Investigator Award, both awarded to J.S.B.

## CONFLICT OF INTEREST STATEMENT

The authors declare that the research was conducted without any commercial or financial relationships that could represent conflicts of interest.

## ETHICS STATEMENT

The studies involving humans were approved by The Ohio State University Biomedical Sciences Institutional Review Board. The studies were conducted in accordance with local legislation and institutional requirements. The human samples used in this study were acquired from an existing biorepository that collects and stores human biosamples and data from critically ill patients at Ohio State University. The biorepository project utilizes broad consent to collect and store samples for use in secondary analyses. Written informed consent for participation was not required from the participants or the participants' legal guardians/next of kin in accordance with national legislation and institutional requirements.

## Data Availability

The data presented in the study will be deposited in the Gene Expression Omnibus (https://www.ncbi.nlm.nih.gov/geo/), accession number GSE283501 (https://www.ncbi.nlm.nih.gov/geo/query/acc.cgi?acc=GSE283501).
